# Is Cesarean Section Ever Justifiable in the Management of Placenta Previa?

**Published:** 1905-08

**Authors:** Richard Douglas

**Affiliations:** Nashville, Tenn.


### SELECTION.

### Is Cesarean Section Ever Justifiable in the Management of Placenta Previa?

By RICHARD DOUGLAS, M. D., Nashville, Tenn.

[From *American Medicine*, January 21, 1905.]

LAWSON TAIT'S brief article in the *Medical Record*, in 1899, practically initiated the question of the justifiability of cesarean section in the management of placenta previa. His advocacy was such a radical departure from the ordinary obstetric methods that the profession was loath to accept it.

With the appearance of E. Gustav Zinke's essay, read before the American Association of Obstetricians and Gynecologists, September, 1901, the question became a warm one.

From the time of the publication of Tait's paper in 1899 to April, 1903, Truesdale was able to collect only 13 recorded cases of cesarean section, done for placenta previa. This shows with what indifference the suggestion was received. But within the last year, owing to the effective advocacy of Zinke, this number has doubled. Evidently, in a limited way, this radical departure is meeting with endorsement. It is yet an open question which Ehrenfest's statistic argument has not answered in the negative; nor has the logical discourse of Higgins restrained the hand of the surgeon; nor yet does the oft-quoted statement obtain that "surgeons who advocate this step have had little or no experience in obstetrics," for I will show that under proper conditions it receives the endorsement of many of our leading obstetricians.

In seeking an answer to this very important question, results prior to the aseptic era should not be used against the obstetric methods. Modern methods alone are under consideration. Two lives are quivering in the balance. To the mother we must concede every right of our first consideration. It is certainly at variance with all human instincts entirely to disregard that of the child; yet, it is axiomatic in obstetric practice that in the management of placenta previa, that which is best for the mother is opposed to the interest of the child. Now, if under proper conditions cesarean section can be shown to be in the interest of both mother and child, surely a substantial advance over the canonic methods is established.

We shall attempt a solution of this subject through a study of methods and results. De Lee, at his last report, had 30 cases of placenta previa without a maternal death; Fry was equally successful in 14. Hirst collected 104 cases, with the death of only one mother. These exceptional records are in striking discord with the results usually obtained.

In the Sloan Maternity, in New York, the maternal mortality is 12 per cent. At the Boston Lying-in Hospital, in 75 cases, Higgins found a maternal mortality to be 10.16 per cent., and upon the basis of these figures, he argues against cesarean section. Strassmann, in a series of 231 cases of placenta previa, found a maternal mortality of 9.25 per cent. Statistics without number may be introduced in evidence that the mortality of placenta previa, unclassified, is fully 10 per cent. This in the hands of our obstetric masters; it would be very safe to claim a much greater mortality in the private work of the general practitioner. I have had 12 cases of placenta previa in which treatment was followed by obstetric methods, losing two mothers.

No one has had the temerity to recommend sweepingly cesarean section for all cases of placenta previa. The most enthusiastic advocates of the operation restrict it to placenta previa centralis; and in this only when there are complicating conditions. Therefore, it is interesting to see what the maternal deathrate is in those cases with central attachment of the placenta, treated by the accepted methods. In this investigation we are met by difficulties; for, unfortunately, very few records give a classification of the variety of placenta previa, and for our argument, everything depends upon this.

According to the figures of Heffmeyer, Behm, Lomer and Strassmann, a prognosis is from three to eight times more serious in central previa than in other varieties (Williams). This gives a range of mortality from 9 per cent. to 30 per cent.

Dorland's figures are valuable for our purpose. He collects 88 cases of central attachment of the placenta. In these the maternal mortality was 22 8-11 per cent. Schauta finds that in 50 cases of placenta previa centralis, 18 per cent. of the mothers was lost.

If it were possible to ascertain from the records it would be interesting to know how many of these cases of placenta previa are met in primiparas. A contemplation of the mortality would, doubtless, be appalling.

After this review of the maternal deathrate, an inquiry into that of the child naturally follows. The modern obstetric methods, especially the application of aseptic technic, have greatly reduced the maternal mortality; it has not materially affected the infantile. Hirst places it at 50 per cent. At the Sloan Maternity it was 45 per cent. In 251 cases of Strassmann, it was 61 per cent.; in Schauta's 254 cases it was 70 per cent. De Lee, in 25

cases, saved all the mothers, but is "strangely silent" in regard to the children. I cannot comprehend the assertion of Grandin and Jarmin, that 90 per cent. of the children should be saved. Fry, in his 14 cases, in which all the mothers were saved, lost 9 children.

These figures are all based upon unclassified placenta previa. In a series of cases of placenta previa one may expect a maternal mortality of 10 per cent. and a fetal mortality of 50 per cent., and may comfortably congratulate himself if these results are obtained. In central attachment of the placenta it is not unfair to estimate the maternal mortality at 20 per cent., and in such cases 75 per cent. to 85 per cent. of the children are lost. Erich Radtke[1] pursues the study of placenta previa beyond the puerperium, and in a study of 80 patients who had suffered from placenta previa, 30 per cent. were sterile; 23 per cent. had aborted at subsequent pregnancies; 57 per cent. of the 80 patients suffered from subsequent anemia, vertigo, headache, and the like. They were found to be suffering from various pathologic conditions, such as endometritis, laceration of the cervix, perineum, and the like. From the facts obtained from these studies Radtke concludes that in very many cases marked injury to the health results from placenta previa.

Now, what of cesarean section. Not for placenta previa, but the elective operation, when, performed for absolute conditions. Higgins states that the mortality still remains about 25 per cent. Hirst affirms this, and Harris says the American mortality for cesarean section is about 30 per cent.

There have been 32 cases in the last five years in the Lying-in Hospital in Boston, with a mortality of 10 per cent. In 551 cases of cesarean section collected by Olshausen and Veit the mortality was 19 per cent. Opposed to this, we have the personal cases of Olshausen, Leopold, and Zweifel, with mortality of 3 per cent. The remarkable record of Reynolds, of Boston, with 22 successive successful cases, and the well known excellent results of Ill, Hirst, Kelly, and others, show that a well-conducted cesarean section upon a fit subject is a comparatively safe procedure.

Insufficient as my own experience has been, it nevertheless has weight with me. I have had 3 cesarean sections and 3 Porro operations on women, at or near full term. All 6 of the mothers recovered. Only 3 of the children were known to be alive at the time of operation, 2 of these were saved and are now living. Two of these patients were operated upon in log huts, in remote country districts, at night, with insufficient light and meager assistance, amid surroundings in every way unfavorable. Both of these

1. Centralblatt für Gynäkologie, November 19, 1903.

patients recovered. Upon this limited experience, I think Deaver is approximately correct when he says that "cesarean section for other conditions than placenta previa, has a mortality of about 10 per cent."

In what does cesarean section for placenta previa differ from the elective operation? There are several reasons that contribute to a higher mortality in the placenta previa cases: 1. It is an operation of emergency; sufficient time cannot be granted for proper preparation. 2. The patient may have bled freely. 3. In all probability she has been subjected to repeated examinations and thus exposed to infection. These all serve to increase the maternal mortality. These factors, separately or combined, may constitute absolute counterindication to operation.

Deaver, whose statistics are the latest that I have seen, finds that in the 24 recorded cases of cesarean section for placenta previa there was a maternal mortality of 20 per cent. An analysis of these cases shows that in many instances the operation was undertaken in the face of conditions which should have been prohibitive.

If, from the list of 24 cases of cesarean section for placenta previa, we select the patients that were proper subjects for the operation, we find the maternal mortality reduced to 18.75 per cent. By cesarean section in these cases, the fetal mortality is reduced to 56 per cent. These are the results in placenta previa with rigid cervices and other unfavorable conditions. Comparing this with the known fetal death rate in placenta previa centralis, 75 per cent. to 85 per cent., we have a saving of 30 per cent. in lives. This, in itself, is sufficient to warrant a sober consideration of the propriety of the operation. Certainly it is not our right, in the interest of the child, to imperil the mother; neither is it our privilege entirely to disregard the child. Yet, we cannot hope that cesarean section for placenta previa, will ever yield the low infantile mortality that is achieved in the elective cesarean section for pelvic deformities, and the like. We must not lose sight of the fact that in 62 per cent. of the cases of placenta previa the patients are prematurely delivered, and the mortality of premature children is known to be very high. This argument is neither for nor against the surgical over the obstetric methods; and while it is held by some that a child delivered through the abdomen does not breathe well, good authorities deny this, and I have not specially observed it. In effecting dilation of the uterus, sufficient even for bipolar version, the placenta is more or less separated and the asphyxiated child encounters greater peril, even if quickly extracted through the parturient canal, than confronts it by the cesarean route.

Briefly summarised, the argument stands about in this light. The maternal mortality of placenta previa centralis, treated obstetrically, varies from 18 per cent. to 30 per cent.; and as Radtke has shown, many who recover sustain pelvic lesions, resulting in invalidism. Against this, when the same condition is treated by cesarean section, the mortality is 18.75 per cent. (Deaver.)

The infantile mortality in placenta previa centralis, by vaginal delivery, is 75 per cent. to 85 per cent. In properly selected cases, it is only 56 per cent. after cesarean section. These are the actual figures, justified by the results up to date. It is my firm conviction that the maternal mortality can be reduced below 10 per cent. We are not warranted in anticipating a reduction in the fetal mortality below 50 per cent.

My arguments are not for the universal adoption of cesarean section in the management of placenta previa. All obstetricians are not capable of doing abdominal surgery, neither are all obstetricians qualified to dilate dextrously a resistant cervix, guard against infection and hemorrhage, securely place the colpeurynter or the gauze tampon, and execute skilful bipolar version; and very few can safely conduct an accouchement forcé. The abdominal surgeon's work is in the open; his mistakes and accidents are revealed. But the obstetrician's are hidden within the deep recesses of the vagina and uterus.

The field of application for cesarean section in these cases is limited, but I believe that the conclusions of Zinke are in the main correct. He says: "I firmly believe that the cesarean section and Porro operations are perfectly legitimate and elective procedures in all cases of placenta previa, central and complete, and especially so when the patient is a primipara, when the os is closed and the cervix unabridged; when hemorrhage is profuse and cannot be controlled by tampons and separation of the placenta around the internal os is difficult or impossible."

I would modify this by saying that cesarean section is indicated in cases of central attachment of the placenta in a primagravida with an undilated and rigid cervix, with moderate hemorrhage, a viable child and the operator a capable abdominal surgeon. The counterindications to the operation are an exsanguinated patient, or one who has been subjected to various obstetric efforts, the presence or probability of infection, a dead fetus and an unskilled operator. The propriety of a Porro or a Duhrssen operation in the presence of infection is another question.

Mrs. T., aged 28, was eight months advanced in her first pregnancy, when, on May 31, at 6.30 p. m., she was seized with a sudden free hemorrhage. There was no pain or other warning symptom preceding or accompanying the loss of blood. Dr. R. O. Tucker was summoned at this time and found her bleeding freely. The cervix was long, conical and rigid. An opiate was administered, the hemorrhage lessened in quantity, but continued moderately until 8 p. m., at which time a rather profuse bleeding again occurred. I saw her in consultation at 9.30 p. m.—three hours after the onset of the symptoms. There was still slight hemorrhage, but the patient was in fair condition. On examination I found a narrow vagina; she was a very compact woman; the cervix was long and rigid, and with some force and difficulty, I managed to insinuate my finger through the cervical canal and recognised a placenta previa centralis. Cesarean section was suggested, to which Dr. Tucker consented. The conditions to my mind seemed ideal. The patient was in good condition, child was living and near full term. Dr. Tucker's examination was careful and cleanly; there had been no attempt to tampon or dilate—hence, the chances of infection were small. It was clear to us all that delivery through the birth canal would be attended by the greatest jeopardy to the mother and almost certain death to the fetus. Knowing that Strassmann found that 34 per cent. of 61 cases in which the patients were treated by tamponade had fever, we deemed it wise to move this patient carefully, without packing, the short distance to my infirmary. She was thoroughly prepared for immediate operation. The classic Sänger cesarean section was done. The lower segment of the uterus was held by the assistant's hands; no tourniquet was used. After the extraction of the child, I was in no haste to remove the placenta. After contraction and retraction of the uterus, the placenta was removed without difficulty or hemorrhage. I then passed my hand into the uterus, dilated the cervix from within, flushed out the uterine cavity with hot saline solution, poured through the incision in the fundus and allowed to pass into the vagina. A wide strip of gauze was placed in the uterus and brought out through the cervix by an assistant. The uterine and parietal wounds were closed in the usual way; the patient was placed in bed in good condition, evincing no shock nor other distress. Neither stimulants nor saline solutions were required. The child cried as it was extracted from the uterus and after a little attention from Dr. Tucker was in good condition. Twenty-four hours after operation a temperature of 100.4° was recorded. The gauze was removed from the uterus and the temperature declined to normal. Milk appeared on the third day, but the quantity was not sufficient to nourish the child. The patient had an afebrile recovery and left the infirmary on the eighteenth day after operation.

Knowing the questionable propriety of cesarean section for placenta previa, and contemplating reporting the case before our local society, I wished to obtain the views of some of the leaders of obstetric and surgical thought upon this question, I sent the following telegram to 14 different men:

"In placenta previa centralis; first pregnancy; child viable; compact woman; rigid cervix; free hemorrhage; surroundings favorable; would you endorse cesarean section? Kindly answer."

With the following answers, I rest the case:

"Conditions justify such action; best chances for mother and child."—Henry D. Fry.

"If child living do immediate cesarean section, otherwise forcible delivery for mother's sake."—W. R. Pryor.

"If you do cesarean section, put large drain of strong iodoform gauze from uterus down through cervix."—W. R. Pryor.

"Yes, would do cesarean section in interests of both mother and child, if very difficult to use colpeurynter."—Howard A. Kelly.

"I unhesitatingly advise cesarean section."—George H. Noble.

"Cesarean section if cervix cannot be dilated without great danger; otherwise not."—J. Whitridge Williams.

"Believe cesarean section safest under conditions named, if patient not exsanguinated."—Charles P. Noble.

"Cesarean section offers best chance of recovery."—Charles A. L. Reed.

"By all means, yes; have had a successful and easy case only lately."—Edward J. Ill.

"Yes, if surroundings are favorable, and if you will do the work."—L. H. Dunning.

"Certainly, if cervix very rigid."—Clarence J. Webster.

"Not as a rule, unless cervix very rigid, hemorrhage more than average, and version seems difficult."—B. C. Hirst.

"I do not favor cesarean section under the circumstances stated."—E. S. Lewis.

